# Analysis of Choroidal Vascularity in Children with Unilateral Hyperopic Amblyopia

**DOI:** 10.1038/s41598-019-48613-3

**Published:** 2019-08-21

**Authors:** Jiwon Baek, Anna Lee, Miyoung Chu, Nam Yeo Kang

**Affiliations:** 1 Department of Ophthalmology, Bucheon St. Mary’s Hospital, College of Medicine, The Catholic University of Korea, Gyeonggi-do, Korea; 2 Department of Ophthalmology, Catholic Medical Center, College of Medicine, The Catholic University of Korea, Seoul, Korea

**Keywords:** Vision disorders, Diagnostic markers, Retina

## Abstract

This institutional case control study was carried out to compare choroidal vascularity (CV) in amblyopic eyes, fellow eyes, and control eyes in children with unilateral hyperopic amblyopia. Sixty-four eyes of 32 childeren with unilateral anisometropic hyperopic amblyopia and 38 eyes of 19 healthy children (controls), aged 3 to 16 years. Subfoveal choroidal thickness (CT) and CV were measured using spectral domain optical coherence tomography. The mean subfoveal CT of amblyopic eyes (338.9 ± 60.0 μm) was greater than that of fellow eyes (315.3 ± 63.3 μm, *P* = 0.043) and control eyes (313.0 ± 42.1 μm, *P* = 0.025). The mean CV of amblyopic eyes (0.715 ± 0.020) was greater than that of control eyes (0.700 ± 0.020, *P* < 0.001). While a positive correlation between CT and CV was found in normal eyes (*r* = 0.470, *P* = 0.004), a strong negative correlation existed in amblyopic eyes (*r* = −0.684, *P* < 0.001). In conclusion, although mean CV was higher in amblyopic eyes, the negative correlation between CT and CV may suggests insufficient blood supply to the outer retina and choroid in the affected eyes of patients with unilateral anisometropic hyperopic amblyopia.

## Introduction

Amblyopia is a disorder that shows subnormal visual acuity (VA) and contrast sensitivity in one or both eyes, which is caused by either visual deprivation or abnormal binocular interactions^1^. It is the most common cause of unilateral vision impairment in children and young adults, and the incidence is reported to range 1–3.5%^2^. The main causes are anisometropia, strabismus, or a combination of both. Amblyopia can involve different levels of the visual pathway such as the extensive visual cortex, lateral geniculate nucleus, and retina^3–5^.

Possible effects of amblyopia on the choroid are currently being investigated. The choroid has been shown to be associated with development of the refractive state and axial elongation in experimental animal models^6^. Additionally, introduction of enhanced depth imaging optical coherence tomography (EDI-OCT) has enabled *in vivo* cross imaging of choroid tissue^7^. In recent years, numerous studies have used EDI-OCT technology to evaluate choroidal thickness (CT) in amblyopia. A meta-analysis of unilateral amblyopia reported that CT increased in amblyopic eyes^8^.

More recently, a methodology has been introduced to evaluate choroidal vascular area by calculating the ratio of vascular luminal area to total choroidal area^9–11^. This quantitative measurement of choroidal vascularity (CV) has also enabled qualitative assessment of choroidal components using choroidal thickness alone, something that was previously not possible. This methodology could be used to generate more detailed information about how choroidal change is involved in the pathogenesis of amblyopia. In this study, we analyzed changes in CT and CV and correlation between them in children with anisometropic hyperopic amblyopia and compared between amblyopic eyes, fellow eyes, and normal controls.

## Results

### Baseline characteristics

In total, 32 pediatric patients with anisometropic hyperopic amblyopia (32 amblyopic eyes and 32 fellow eyes) and 19 normal control patients (38 eyes) were included in the study. Mean patient age was 6.9 ± 2.9 years (range; 3–12 years) and 7.3 ± 2.3 years (range; 3–16 years) for the hyperopic amblyopia group and control group, respectively (*P* = 0.607). Mean refractive error, BCVA, and AL differed between amblyopic eyes, fellow eyes, and control eyes (all *P* < 0.001) (Table 1).

**Table 1:** Comparison of demographic and clinical parameters between groups.

Parameters	Unilateral amblyopic children		Controls	p-value^a^	p-value^b^	p-value^c^	p-value^d^
	Amblyopic eyes	Fellow eyes					
No. eyes (patients)	32 (32)	32 (32)	38 (19)				
Age, years, mean ± SD (range)	6.9 ± 2.9 (3–12)		7.3 ± 2.3 (3–16)	0.607	0.233	0.233	
Male, n (%)	15 (47)		14 (37)	0.403			
Refractive error, D, mean ± SD	4.16 ± 1.97	2.32 ± 2.37	−0.75 ± 1.35	0.039**	<0.001*	<0.001*	<0.001*
BCVA, LogMAR, mean ± SD	0.30 ± 0.22	0.13 ± 0.24	0.02 ± 0.05	<0.001**	<0.001*	0.014	<0.001*
Axial length, mm, mean ± SD	21.28 ± 1.05	22.25 ± 0.87	23.38 ± 0.79	<0.001**	<0.001*	<0.001*	0.002*

SD: standard deviation; D: diopter; BCVA: best-corrected visual acuity; logMAR: minimal angle of logarithm. ^a^Kruskal Wallis test between Amblyopic eyes, fellow eyes, and controls. ^b^Mann-Whitney test between amblyopic eyes and controls. ^c^Mann-Whitney test between fellow eyes and controls. ^d^Mann-Whitney test between amblyopic eyes and fellow eyes. *Statistically significant p-value.

### Comparison of CT and CV between groups

Intraclass coefficients for nasal, subfoveal, and temporal CT and CV were 0.899, 0.901, 0.885, and 0.879, respectively. The mean CT and CV at all three points (nasal, subfoveal, and temporal) differed between amblyopic eyes, fellow eyes, and control eyes (*P* < 0.001, 0.023, 0.049, and 0.020, respectively). The mean CT at all three points was higher in amblyopic eyes compared to both fellow eyes and control eyes (p = 0.023 and *P* = 0.013 for nasal CT, 0.043 and 0.025 for subfoveal CT, and 0.021 and 0.030 for temporal CT). There was no difference between fellow eyes and control eyes for nasal, subfoveal, or temporal CT (*P* = 0.837, 0.930, and 0.976, respectively). Mean CV was higher in amblyopic eyes compared to normal eyes (*P* < 0.001), but no significant difference was found between amblyopic eyes and fellow eyes (*P* = 0.511). Mean CV in fellow eyes was higher than that in control eyes (*P* = 0.006) (Table 2).

**Table 2:** Comparisons of choroidal thickness and vascularity between groups.

	Amblyopic eyes (n = 32)	Control eyes (n = 38)	Fellow eyes (n = 32)	p-value^a^	p-value^b^	p-value^c^	p-value^d^
CT nasal, μm, mean ± SD	314.9 ± 55.4	286.2 ± 39.4	289.2 ± 59.3	0.023*	0.013*	0.837	0.023*
CT subvfoveal, μm, mean ± SD	338.9 ± 60.0	313.0 ± 42.1	315.31 ± 63.3	0.049*	0.025*	0.930	0.043*
CT temporal, μm, mean ± SD	317.1 ± 58.3	288.5 ± 44.6	292.1 ± 54.3	0.020*	0.011*	0.976	0.021*
CV, mean ± SD	0.715 ± 0.020	0.700 ± 0.020	0.710 ± 0.024	0.001*	<0.001*	0.006*	0.511

CT: choroidal thickness; CV: choroidal vascularity; SD: standard deviation. ^a^Kruskal Wallis test between Amblyopic eyes, fellow eyes, and controls. ^b^Mann-Whitney test between amblyopic eyes and controls. ^c^Mann-Whitney test between fellow eyes and controls. ^d^Mann-Whitney test between amblyopic eyes and fellow eyes. *Statistically significant p-value.

### Correlation analysis

**Figure 1:**
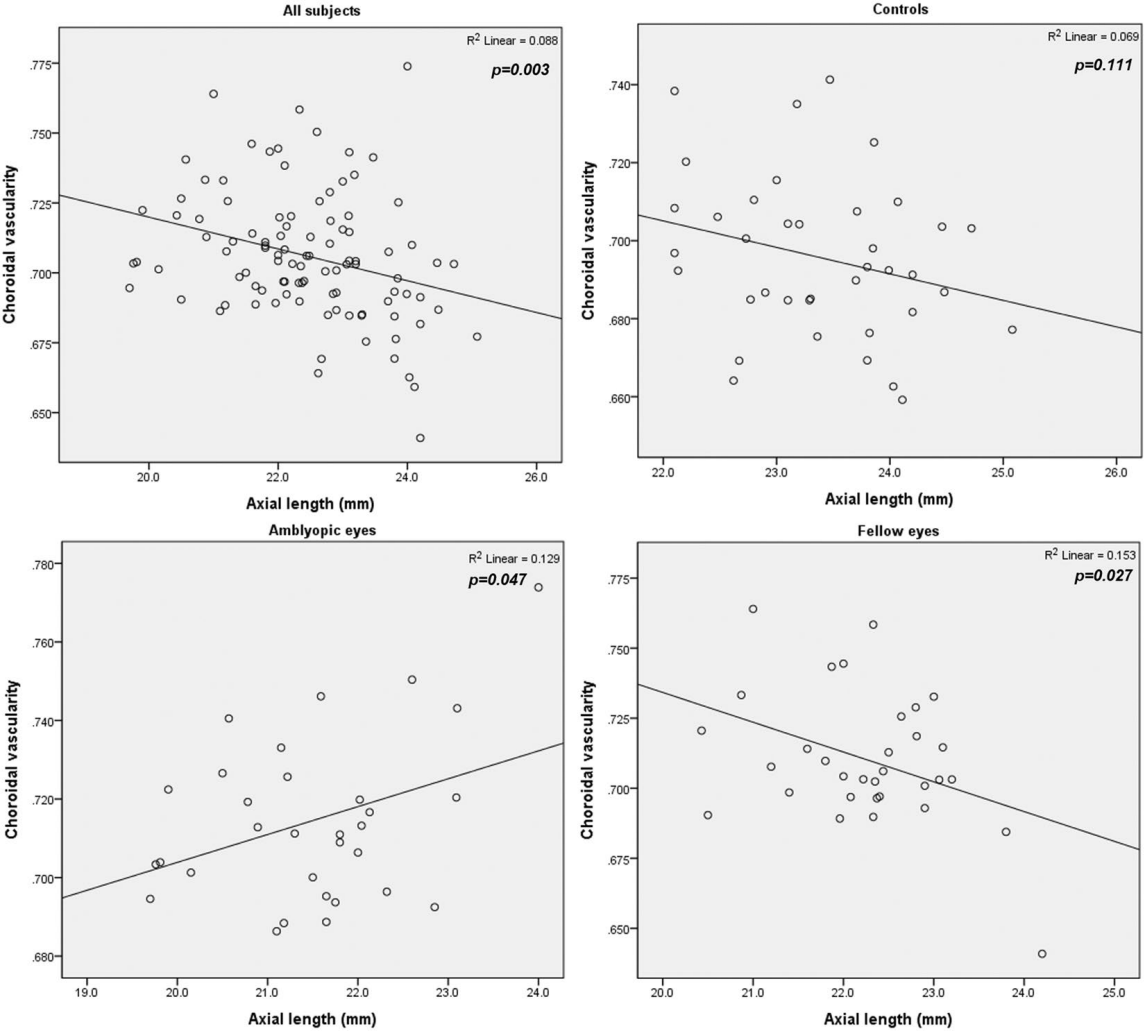
Correlation between axial length and choroidal vascularity. Averaged for all eyes in the study, choroidal vascularity (CV) had a negative correlation with axial length (AL) (*r* = −0.297, *P* = 0.003). In both groups, CV positively correlated with AL in amblyopic eyes and negatively correlated in fellow eyes (r = 0.360, *P* = 0.047; and *r* = −0.391, *P* = 0.027).

**Figure 2:**
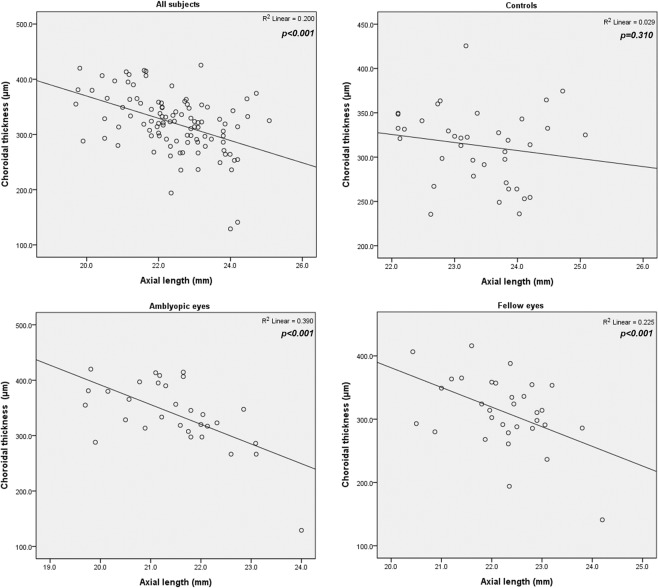
Correlation between axial length and subfoveal choroidal thickness. Subfoveal choroidal thickness had a negative correlation with axial length when all eyes were analyzed together (*P* < 0.001) and in amblyopic eyes and fellow eyes (both *P* < 0.001).

For correlation analysis of all eyes used in this study, CV revealed a negative correlation with AL (*r* = −0.297, *P* = 0.003) and positive correlations with refractive error (r = 0.384, *P* < 0.001) and BCVA (r = 0.290, *P* = 0.003) (Table 3). This correlation trend did not hold for analyses run for each group separately: CV positively correlated with AL in amblyopic eyes (r = 0.360, *P* = 0.047) and negatively correlated with AL in fellow eyes (*r* = −0.391, *P* = 0.027) (Fig. 1). CT negatively correlated with AL when all eyes were grouped together (*P* < 0.001) as well as in amblyopic eyes and fellow eyes (both *P* < 0.001) (Fig. 2).

**Table 3:** Correlation between choroidal vascularity and clinical parameters.

Parameters		All eye (n = 102)	Amblyopic eyes (n = 32)	Controls (n = 38)	Fellow eyes (n = 32)
Age	Pearson Correlation	0.005	0.266	−0.205	−0.194
	p-value	0.963	0.140	0.216	0.296
AL	Pearson Correlation	−0.297*	0.360*	−0.263	−0.391*
	p-value	0.003	0.047	0.111	0.027
BCVA	Pearson Correlation	0.290*	0.275	0.004	0.084
	p-value	0.003	0.128	0.981	0.648
RE	Pearson Correlation	0.384*	0.142	0.286	0.172
	p-value	<0.001	0.438	0.081	0.348

VA: visual acuity; BCVA: best-corrected visual acuity; RE: refractive error; AL: axial length. *Correlation is significant at the 0.05 level (2-tailed).

**Figure 3:**
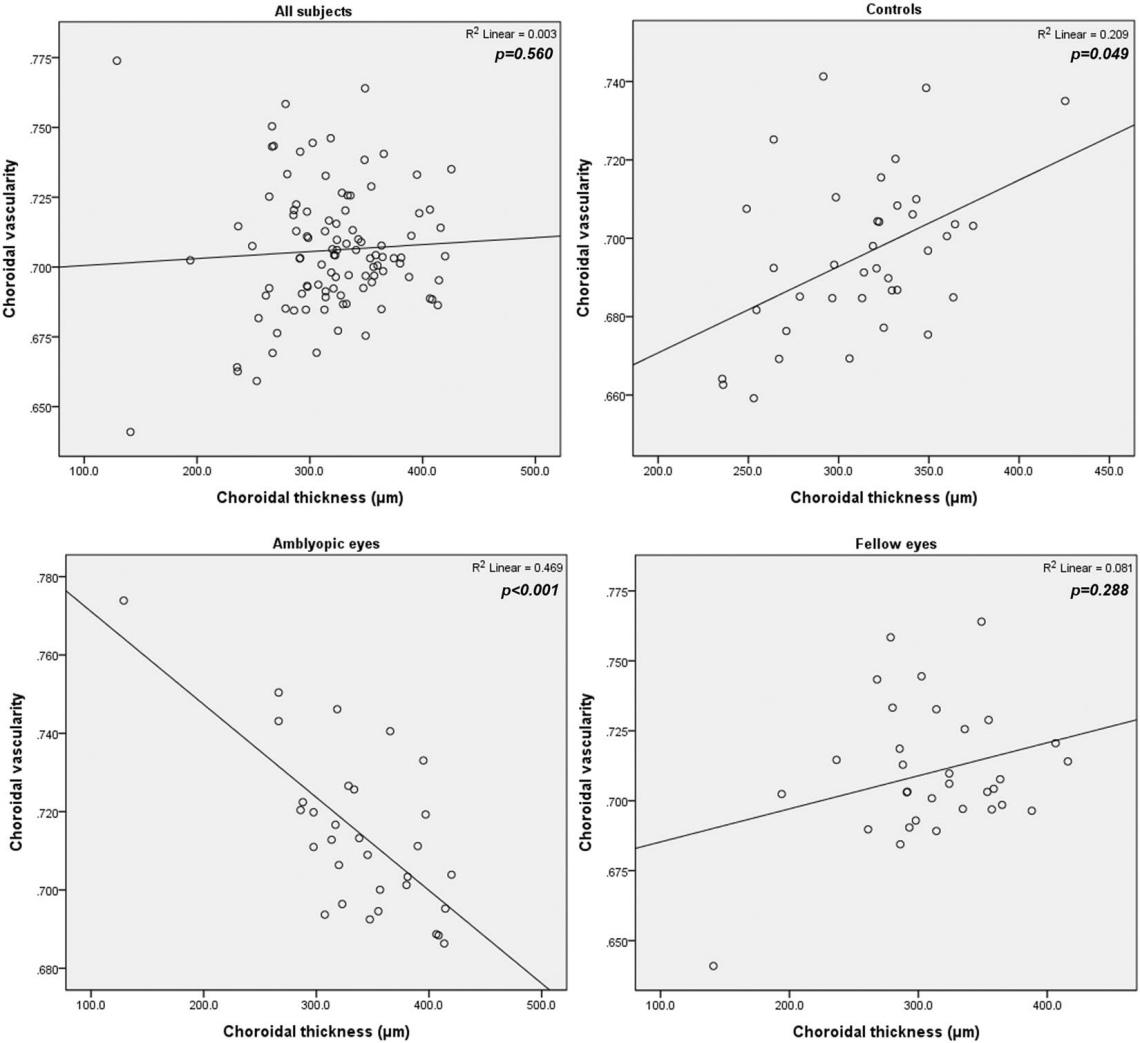
Correlation between choroidal vascularity and subfoveal choroidal thickness. In all eyes analyzed together, no significant correlation was measured (*P* = 0.560). In amblyopic eyes, a negative correlation between subfoveal choroidal thickness and choroidal vascularity was found (*r* = −0.684, *P* < 0.001), whereas a positive correlation was found in normal eyes (r = 0.47, *P* = 0.004).

For the correlation analysis between CT and CV, no significant correlation was found when including all eyes (*P* = 0.500, 0.560, and 0.644 at nasal, subfoveal, and temporal, respectively). However, correlation analysis for each group yielded different results. In amblyopic eyes, a strong negative correlation between subfoveal CT and CV was found (*r* = −0.684, *P* < 0.001). In contrast, a positive correlation was found in normal eyes (r = 0.47, *P* = 0.004). The Pearson coefficient for fellow eyes was 0.301 but was not statistically significant (*P* = 0.099) (Fig. 3). The correlation results between CT and CV were identical for nasal and temporal CT (Table 4).

**Table 4:** Correlation between choroidal thickness and choroidal vascularity in each group.

		Amblyopic eyes				Controls				Fellow eyes			
		CT nasal	CT foveal	CT temporal	CV	CT nasal	CT foveal	CT temporal	CV	CT nasal	CT foveal	CT temporal	CV
CT nasal	Pearson Correlation	1	0.967^**^	0.947^**^	−0.659^**^	1	0.839^**^	0.693^**^	0.339^*^	1	0.932^**^	0.916^**^	0.243
	p - value		0.000	0.000	0.000		0.000	0.000	0.037		0.000	0.000	0.180
CT subfoveal	Pearson Correlation	0.967^**^	1	0.946^**^	−0.684^**^	0.839^**^	1	0.891^**^	0.392^*^	0.932^**^	1	0.930^**^	0.198
	p - value	0.000		0.000	0.000	0.000		0.000	0.015	0.000		0.000	0.277
CT temporal	Pearson Correlation	0.947^**^	0.946^**^	1	−0.657^**^	0.693^**^	0.891^**^	1	0.321^*^	0.916^**^	0.930^**^	1	0.194
	p - value	0.000	0.000		0.000	0.000	0.000		0.049	0.000	0.000		0.288
CV	Pearson Correlation	−0.659^**^	−0.684^**^	−0.657^**^	1	0.339^*^	0.392^*^	0.321^*^	1	0.243	0.198	0.194	1
	p - value	0.000	0.000	0.000		0.037	0.015	0.049		0.180	0.277	0.288	

CT: choroidal thickness; CV: choroidal vascularity. **Correlation is significant at the 0.01 level (2-tailed). *Correlation is significant at the 0.05 level (2-tailed).

## Discussion

Previous studies reported an association between amblyopia and choroidal thickness, with most studies suggesting that the choroid is thicker in amblyopic eyes than in normal eyes. The choroid is composed of abundant blood vessels surrounded by stromal tissue which is comprised of connective tissue, melanocytes, nerves, and extracellular fluid^12^. Because the role of choroid tissue in the pathogenesis of amblyopia is likely related to its role in providing blood supply to the outer retina, analysis of blood flow is more important than measurement of choroidal thickness alone. This study is important because it focuses on the vascularity of the choroid, a highly relevant metric for understanding blood flow. This comparative study involving 32 amblyopic eyes, 32 fellow eyes, and 38 normal control eyes revealed that choroid was thicker and choroidal vascularity was higher in amblyopic eyes compared to the controls.

Mean CT was greater in amblyopic eyes than in fellow and control (normal) eyes, and no significant difference in CT was found between fellow and control eyes. These results align with previous reports^8^. In studies that compared CT of amblyopic eyes to CT of fellow and age-matched control eyes in anisometropic hyperopic children^13–16^, the weighted mean difference of CT between amblyopic eyes and fellow eyes was 57.69 (95% confidence interval (CI) 36.71–78.68), and the weighted mean difference of CT between amblyopic eyes and control eyes was 55.65 (95% CI 19.37–91.2)^8^. In the present study, the differences were 23.59 and 25.90, respectively. These values are near the lower limits of previous studies. Deviations in data presented here and previously reported data may be due to difference in the OCT system used—all 4 previous studies used a Spectralis system, whereas the current study used a Cirrus system—or age differences between the patients used in the studies. Nevertheless, a significant increase of CT in amblyopic eyes compared to fellow and control eyes was confirmed in this study.

Mean CV of amblyopic eyes was higher than that of control eyes. In healthy eye studies, greater CT is associated with higher CV^9^. The positive correlation between CT and CV was also evident in normal subjects in the study. Higher mean CV in amblyopic eyes may lead to increased CT. Additionally, there are some reports that central macular thickness is higher in amblyopic eyes than in fellow or normal eyes^17–20^. In that regard, increased CT may be a consequence of increased blood flow due to increased blood supply requirement to the thickened macula, especially when foveola is composed of outer retina only (i.e. lacks blood supply from retinal vessels) and has to be nourished by the choroidal vasculatures^8^. The result of this study further supports the notion of increased blood flow in the amblyopic eyes since higher CV is indicative of increased vascular component in the choroid.

In total eyes, CT showed negative correlation with AL. Mean CV of fellow eyes did not differ from that of amblyopic eyes and this may be attributable to the general trend that CV increases with shorter axial length. In terms of refractive errors and axial length, fellow eyes revealed significant hyperopic refractive error and shorter axial length compared to control eyes. On the other hand, the correlation between CV and AL was not consistent between groups. While the correlation trend between CV and AL was negative in fellow eyes and control eyes, CV tended to decrease with shorter AL in amblyopic eyes. This means that, in amblyopic eyes, even though CT got thicker with shorter AL, CV did not increase accordingly. This is of particular interest because insufficient blood supply to the outer retina and choroid could be the result of lower vascularity in thicker choroid in amblyopic eyes. Therefore, while we cannot determine if it is a causal or resultant phenomenon, we suggest decreased choroidal blood flow as a possible mechanism for amblyopia in anisometropic hyperopic eyes.

Another theory explaining an increase in CT in amblyopic eyes is that the choroidal changes in CT in response to defocus did not occur in the hyperopic amblyopic eyes^14^; in this case, subfoveal CT would be thicker and ocular growth would be limited. This process is thought to involve the non-vascular smooth muscle cells (NVSMC) of the choroid, which are located in the stroma^21,22^. In this case, it can be hypothesized that higher vascularity in an amblyopic choroid may exert more resistance to choroidal thinning caused by NVSMCs, and the compensation mechanism used for defocusing does not function in these eyes. The eye thus becomes amblyopic. On the other hand, high vascularity itself may be caused by lack of a stromal component. In that case, compensation to defocus may not be possible due to either a complete lack or decreased number of NVSMCs. Again, that can lead the eye to become amblyopic.

Although we used an EDI mode of spectral domain OCT, which is widely used to study choroid tissue, our method has some limitations, including manual measurement and difficulty of distinguishing the chorioscleral border due to irregularities. To overcome these limitations, we used measurements of CT and CV from two different observers (ICCs were fairly high). However, we believe that validation of this study is required with other methods, since the visualization of choroid can be improved by using other OCT systems (e.g., swept-source OCT, which is known to have better resolution for choroid tissue). Future studies with larger sample sizes would also be warranted to validate this study. Additionally, this study only looked at the place where the b-scan intersects the subfoveal area. The comparisons of choroidal thickness and vascularity could include a wider area.

In conclusion, the mean CV of amblyopic eyes was higher than that of the controls. In amblyopic eyes, CV decreased with greater CT and shorter AL. These correlations were distinctive from other groups. These results suggest that CV may not increase directly accordingly to the increased with CT in amblyopic eyes even though the overall CV was higher in amblyopic eyes. Further studies are required to confirm and build on our results.

## Methods

This is an observational case control study. We conducted a review of the medical records of pediatric patients with anisometropic hyperopic amblyopia who visited Bucheon St. Mary’s Hospital of The Catholic University of Korea between November 2017 and August 2018. The study was approved by the Institutional Review Board of the hospital, which waived the written informed consent because of the study’s retrospective design and was conducted in accordance with the tenets of the Declaration of Helsinki.

### Patients

Patients aged between 3 and 16 years with anisometropic hyperopic amblyopia in one eye were included. An eye was classified as amblyopic when the best-corrected visual acuity was ≤20/30 or when the eye was at least 2 Snellen chart lines worse than its fellow. Anisometropia was defined as a difference in refractive error between the eyes, in any meridian, >1.0 diopter. Eighteen age-matched controls who had 20/25 visual acuity and no ocular or systemic disease were recruited as normal controls. Exclusion criteria were history of any ocular disease or any systemic disease with ocular findings, history of previous intraocular surgery or laser therapy that could have affected choroidal thickness, and use of any systemic medicine during the previous month.

Comprehensive ocular examinations, including best-corrected visual acuity (BCVA) testing, fundus examination with slit-lamp biomicroscopy, cycloplegic refraction via auto-refractometry (Canon RK-I autorefractometer; Canon, Tokyo, Japan), spectral-domain OCT (SD-OCT) imaging (Cirrus-HD 4000, Carl Zeiss Meditec, Jena, Germany) with an EDI protocol, and axial length (AL) (IOL master, Carl Zeiss Meditec, Dublin, CA) were assessed for all patients. Snellen visual acuity was converted to logarithm of the minimal angle of resolution (logMAR) for statistical analysis. Central macular thickness of the central 1 mm zone was measured automatically using a Cirrus OCT system.

### Choroidal thickness and vascularity measurements

**Figure 4:**
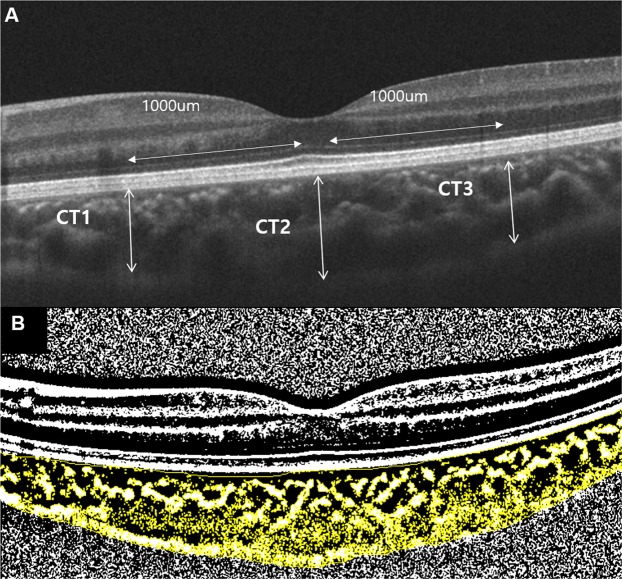
Measurements of choroidal thickness and vascularity. (**A**) Nasal parafoveal choroidal thickness (CT1), subfoveal choroidal thickness (CT2), and temporal parafoveal choroidal thickness (CT3) were obtained using enhanced depth image optical coherence tomography. CT was measured as the distance between Bruch’s membrane and the choroid-scleral interface. (**B**) The subfoveal choroidal area with a width of 3 mm, centered at the fovea, was selected. Using ImageJ software, the image was binarized with Niblack’s method, and the ratio of vascular area (black pixels) to stromal area (white pixels) was quantified.

Only good quality scans, defined as scans with signal strength ≥7 without involuntary saccade or blinking artifacts were used for analysis. All scans were taken between 10 AM and 3 PM. Two independent graders (M.Y.C. and A.L.) performed CT and CV measurements of the choroid. The average measurements of the two observers were used for analysis. Choroidal thickness, defined as the vertical distance between the hyperreflective line of Bruch membrane and the choroidoscleral border, was measured at 3 points using the horizontal line scan intersecting the center of the fovea. Measurements were performed at the fovea and 1000 um from the fovea nasally and temporally (Fig. 4A)^23^. CV at the 3000 μm area centered at the fovea in the same OCT line scan was assessed using a previously described method by Sonoda and associates^11^. Binarization of the transverse cross sectional subfoveal OCT image was measured using a modified Niblack method in Image J (version 1.47; provided in the public domain by the National Institutes of Health, Bethesda, MD, USA; http://imagej.nih.gov/ij/). The ratio of choroidal luminal area to total choroidal area was calculated and defined as CV (Fig. 4B).

### Statistical analyses

Statistical analyses were performed using SPSS statistical software version 19.0 (SPSS Inc., Chicago, IL, USA). Sample sizes with 80% power and 5% significance were calculated for a cross sectional study for CT based on previous studies^8,24^. Comparisons between groups were conducted using the χ^2^ test for categorical variables and Student’s t test for continuous variables following a normal distribution confirmation carried out with the Kolmogorov–Smirnov test. The Mann-Whitney U test was used when a normal distribution was not confirmed. Pearson’s correlation analysis was used to determine the coefficient of correlation between CT and CV. A *P*-value less than 0.05 was considered statistically significant.

## Data Availability

The datasets generated during and/or analysed during the current study are available from 10.6084/m9.figshare.8002097.
